# Revascularization of Transplanted Pancreatic Islets and Role of the Transplantation Site

**DOI:** 10.1155/2013/352315

**Published:** 2013-09-09

**Authors:** Andrew R. Pepper, Boris Gala-Lopez, Oliver Ziff, A. M. James Shapiro

**Affiliations:** ^1^Clinical Islet Transplant Program, University of Alberta, Edmonton, AB, Canada T6G 2C8; ^2^Department of Surgery, University of Alberta, Edmonton, AB, Canada T6G 2C8; ^3^Medicine and Surgical Oncology, Clinical Islet and Living Donor Liver Transplant Programs, Alberta Innovates-Healthcare Solutions (AIHS), University of Alberta, 2000 College Plaza, 8215-112th Street, Edmonton, AB, Canada T6G 2C8

## Abstract

Since the initial reporting of the successful reversal of hyperglycemia through the transplantation of pancreatic islets, significant research efforts have been conducted in elucidating the process of revascularization and the influence of engraftment site on graft function and survival. During the isolation process the intrinsic islet vascular networks are destroyed, leading to impaired revascularization after transplant. As a result, in some cases a significant quantity of the beta cell mass transplanted dies acutely following the infusion into the portal vein, the most clinically used site of engraftment. Subsequently, despite the majority of patients achieving insulin independence after transplant, a proportion of them recommence small, supplemental exogenous insulin over time. Herein, this review considers the process of islet revascularization after transplant, its limiting factors, and potential strategies to improve this critical step. Furthermore, we provide a characterization of alternative transplant sites, analyzing the historical evolution and their role towards advancing transplant outcomes in both the experimental and clinical settings.

## 1. Introduction

Significant progress has occurred in the outcomes of clinical islet transplantation, reflecting improvements in immunosuppression and preparation of sufficient quantities of highly viable islets for transplantation [1]. Solitary islet transplantation has become an accepted modality to stabilize frequent hypoglycemias or severe glycemic lability in highly selected subjects with poor diabetic control, resistant to standard, intensive, or insulin-pump based therapies [1, 2].

Pancreatic islets are highly vascularized, which is important in their ability to quickly secrete insulin in response to changes in blood glucose. After isolation the reestablishment of blood flow to transplanted islets requires several days to weeks and involves angiogenesis and other complex mechanisms during the remodelling process [3]. 

A decade of research working to improve intrahepatic islet delivery has identified multiple mechanisms that limit islet engraftment and long-term function. This vascular space provides nutritional and physical support for islets, an essential role given that the isolation process strips the islets of their dense vasculature and specialized extracellular matrix [4, 5]. However, the hepatic portal vasculature may be considered a hostile environment that may limit successful islet engraftment and function [6]. As a consequence many investigations in this field have pursued alternative sites of pancreatic islet implantation in order to optimize islet engraftment and function, reduce necessary implantation mass, and decrease immunogenicity [7].

We herein review the process of islet revascularization after transplant, its limiting factors, and potential ways to improve this critical step. We also provide a characterization of the transplant site, analyzing the historical evolution and their role towards transplant outcomes in experimental and clinical settings.

## 2. The Islets of Langerhans

The pancreas is a unique organ which is responsible for orchestrating two independent yet vital processes within in the body, one being nutrient absorption through the release of exocrine digestive enzymes and the second involving glucose homeostasis through the release of endocrine hormones. The acinar cells (exocrine), compromising approximately 98% of the pancreas by mass, are responsible for secreting digestive enzyme into pancreatic ducts, while islets of Langerhans (endocrine) account for the additional 2% of the gland's mass and are responsible for maintaining glucose homeostasis through the synthesis and release of hormones [8].

The islets of Langerhans with the pancreas can be regarded as “microorgans” encompassing approximately 1% of the pancreas. Despite their low volume it is estimated that they receive up to 15% of the pancreatic blood supply and are responsible for the gland's endocrine function [8–10]. Since their initial discovery by Paul Langerhans in 1869 and the deduction of their function by Edouard Laguesse in 1893 [11, 12], innovative worldwide research has provided astonishing insight into the complexities and intricacies of these “microorgans.”

The human pancreas contains approximately 1 million islets in a conglomerate of nearly 2,500 cells each, although the individual size varies considerably [8]. The cellular organization within the islet cytoarchitecture has clear homeostatic benefit. Each islet cluster regardless of shape and size contains alpha (*α*), beta (*β*), delta (*δ*), PP, and epsilon cells that synthesize and release glucagon, insulin, somatostatin, pancreatic polypeptide and ghrelin, respectively, typically in a nutrient-dependent fashion [12].

It is estimated that human *α*-cells account for approximately 30% of the cellular composition of the islets, which secrete glucagon, influencing both glucose and ketone regulation [13–15]. Characteristically, elevated blood glucose levels suppress glucagon section and subsequently stimulate insulin release. Conversely, it is difficult to ascertain whether glucose directly or indirectly acts on the *α*-cells since the paracrine effect of other islet cell types in addition to the autonomic nervous system may influence the secretory suppression. The involvement of *β*-cell and *α*-cell communication with respect to the effects of glucose on glucagon secretion has been more thoroughly elucidated. Interestingly, in the context of a type 1 diabetic, where glucose induced insulin secretion has been abolished, the suppressing ability of glucose on glucagon has also been abrogated [16, 17]. Research studying the paracrine effect has proven difficult to interpret as we lack robust methods for studying the communications of peptides throughout the interstitial space. Both the autonomic nervous system and *δ*-cells may account for additional indirect pathways in which elevated glucose suppresses glucagon secretion. It has been demonstrated that high glucose concentration stimulates the release of somatostatin, a known potent secretory inhibitor of glucagon, from *δ*-cells [8, 18]. Fluctuations, in plasma glucose concentrations can affect the autonomic nervous system, which has the potential for influencing the hormonal sections within the islets. For example, during severe hypoglycemic stress the parasympathetic nervous system becomes activated resulting in the release of epinephrine, without influencing the sympathetic nervous system. However, the impact of the autonomic nervous system on human pancreatic islet function has yet to be fully explained [19].

Pancreatic *β* cells form the bulk of the endocrine cellular content (approximately 60%) within the pancreas and secrete the hormone insulin, a 51-aminoacid anabolic peptide which is essential for regulating glucose homeostasis. When high energy substrates are in excess (i.e., postprandial), insulin triggers cells to stimulate glucose, protein and lipid metabolism in addition to RNA and DNA syntheses. Due to the complexity and multitude of the intracellular pathways involved, the exact mechanism of insulin's action is yet to be fully elucidated. However, it is understood that upon hormone-receptor activation a cascade of covalent enzyme modifications occurs, usually in the form of phosphorylation or dephosphorylation of serine, threonine, or tyrosine residues controlled by a balance of protein kinases and protein phosphatases. Furthermore, allosteric feedback and feedforward regulations are critical enzymatic pathways regulating glucose metabolism. The hypoglycaemic action of insulin is the net result from the uptake of glucose via translocation of glucose transporters (GLUT4) and amino acids, activation of protein synthesis from amino acids, in addition to glycogen and triglyceride syntheses from glucose. Furthermore, insulin inhibits breakdown of triglycerides in adipose tissue and gluconeogenesis in the liver. Saltiel and Kahn's 2001 *Nature* review provides an in-depth perspective pertaining to the pathways that involved insulin signalling, glucose, and lipid metabolism, which is a highly recommendable resource. Insulin's isolation and clinical application by Banting and colleagues are regarded as one of the great medical breakthroughs of the 20th century [20, 21]. In addition to insulin, *β*-cell secretes islet associated polypeptide (IAPP, also called amylin), a 37-amino acid peptide [22]. It is hypothesized that under pathological conditions IAPP molecules polymerize to form intraislet amyloid deposits, a characteristic of type 2 diabetic patients and in cases of insulinoma. Interestingly, in the field of islet transplantation it has recently been described that the inflammation induced by islet amyloid deposits indeed contributes to *β*-cell dysfunction after transplant [23].

The pancreatic *δ*-cells, which amount to less than 10% of the islet, secrete somatostatin a hormone originally isolated from the hypothalamus [14, 24]. This peptide is potent inhibitor of glucagon, insulin, and pancreatic polypeptide [8, 25]. The *δ*-cells resemble neurons containing secretory-granules ending near a capillary suggestive of a focal and possibly paracrine influence [26, 27]. The function of somatostatin released from the islet is still unclear in either the physiologically normal or diabetic pancreas [8].

The pancreatic polypeptide (PP) cells secrete the least studied of the islet hormones, pancreatic polypeptide (PP), and account for less than 5% of the islet cellular composition [14, 27]. PP has been demonstrated to have an inhibitory effect on the exocrine secretions from the pancreas, highlighting the communication between endocrine and exocrine cells within the gland [8].

The recently discovered Epsilon or Ghrelin cells encompass less than 1% of a human islet. They are primarily responsible for the secretion of ghrelin, initially isolated from rat stomach and later localized to human islets [28]. The peptide is thought to be of importance in growth hormone release, metabolic regulation, and energy balance, but its role in islets has yet to be conclusively defined [27].

Based on the diverse cellular composition and complex interactions within the islets of Langerhans, it is evident that glucose homeostasis is a dynamic process involving multiple cell types contained within these unassuming “microorgans.”

## 3. Islet Cytoarchitecture and Microcirculation

The organization of the cellular constituents of an islet may as well have important glucose homeostatic benefits; for instances having insulin producing *β*-cells and glucagon producing *α*-cells in close proximity allows their hormones to be secreted directly into the portal system optimizing their effects on glucose control. Although predominately comprised of endocrine cell, nonendocrine cell shares a portion of the islet structure [9]. Vascular endothelial cells account for the majority of nonendocrine cells within the islet [9, 27]. The remaining cellular components of the islet include nerve fibers, pericytes, macrophages, and dendritic cells [29]. 

It is important to be conscience of the species source of islets when conduction research as the observations may not be relevant to humans, due to the variations that exist in islet cytoarchitecture and insulin composition. For instance, mice, rats, and rabbits have a distinct cytoarchitecture that segregates non-*β*-cells (*α* and *δ*) to the mantel (periphery) of the islet, with *β*-cells residing in the islet core [9, 14, 30]. In species such as horse, non-human primates, humans, and pigs, the islets architecture is reversed having the *β*-cells localized more towards the periphery and *α*-cells and other non-*β* cells more evenly distributed throughout the islet [9, 14].

It has been established that, despite differences in islet architecture, the intraislet vasculature is also reversed so that non-*β*-cell to *β*-cell blood flows remain consistent, supporting the notion that perfusion is central to islet function [9, 31]. These variants between species may in part be attributed to the disparity of the cellular composition. Traditionally, human islets were thought to be compromised of greater than 70% *β*-cells, less than 20% *α*-cells, and approximately 10% *δ*-cells and 5% PP cells, respectively, similar to the composition of rodent islets [27, 32, 33]. More recently, studies have demonstrated that human islets are comprised of proportionally fewer *β*-cells (60%) and contain a large population of *α*-cells (30%), compared to mouse islets, which have historically been viewed as the prototypical islet [13–15]. Therefore the different morphology of human islets must be taken into consideration when using experimental animal models as findings pertaining to islet physiology; vasculature and paracrine activity may be incompatible to the pathophysiology of the human islet. 

For instances the rescinded effect of glucose on *α*-cells is thought to be largely contingent upon elevated concentrations of insulin, carried by the local portal vasculature from the rich *β*-cell core to the islet mantel as evident in the rat pancreas [34]. However due to the species variation, this mechanism may not translate to human islet interactions. Portal system experiments conducted in rats, dogs, and primates demonstrated that the intraislet cellular interactions occur in a downstream fashion from *β*- to *α*- to *δ*-cell direction. Therefore, it appears that *β*-cells may not be diretly exposed to hormones released from *α*- and *δ*-cells, and subsequently *α*-cells are not influenced by secretions of *δ*-cell in the portal system [8]. Thus islet vascular communications do not account for all interactions.

Furthermore, models involving isolated islets may not be physiologically relevant or translational as the isolation process disrupts or destroys intrinsic vascular, neural, and interstitial networks.

A significant factor influencing islet survival and function is the rapid and adequate revascularization of transplanted islets, typically intrahepatically. Delayed and insufficient revascularization can deprive islets of oxygen and nutrients, resulting in islet cell death and early graft failure [35]. It would follow that reestablishment of the vascular bed to the transplanted islets would be important for graft survival. Several studies have indicated that factors such as vascular endothelial growth factor- (VEGF-) A, hepatocyte growth factor (HGF), fibroblast growth factor (FGF), epidermal growth factor (EGF), and matrix metalloproteinase (MMP) are major regulators of islet vascularization [36, 37]. Within the islet of Langerhans the intraislet endothelial cells are responsible for the release of these proangiogenic factors. Recent evidence indicates that the endothelial cells creating new capillaries or vessels within the islet graft arise from various sources. Endothelial cells or capillaries from the transplant recipient, which are recruited into the islet graft, create new islet vascular networks. An alternative vascular source could be the intraislet endothelial cells, which exist in large numbers in isolated islets and may account for up to 40% of the endothelial cells lining capillaries within a revascularized graft [38–40]. Interestingly, functional vessels within a re-vascularized graft are often chimeric, consisting of both endothelial cells from the donor and from the recipient. Intraislet endothelial cells have been shown to survive islet transplantation; however, they rapidly disappear during culture [39, 40]. A paradox exists in the culture of human islets prior to transplant as studies have demonstrated that culturing islets improve their insulin secretory capacity; however this gain in metabolic potency may be at the expense of hindered graft revascularization due to the intra-islet endothelial loss [41]. On average, 15–20% of the islet mass may be lost during culture, and it is unclear whether this same proportion would also be lost if transplanted without culture.

## 4. Islet Revascularization after Transplant

The islet isolation process severs the connections between the islet vasculature and systemic circulation. It results in significant ischemic and mechanical injury, rendering islets more susceptible to posttransplant stresses. Islets are metabolically active and require access to oxygen, glucose, and other metabolites in a hospitable environment [7]. As the revascularization of the transplanted pancreatic islet is not immediate, proximity to a good vascular supply is essential. Most isolated islets are 50–100 *μ*m in diameter, and the capacity for diffusion of the transplanted islet is limited. Ideally, therefore, islets should be transplanted into a site with high oxygen supply [7].

### 4.1. New Angiogenesis

In contrast with whole-organ transplantation, where organ perfusion is quickly reestablished by reconnection of arterial and venous vessels, the reestablishment of blood flow to transplanted islets requires several days and involves angiogenesis and possibly vasculogenesis. The death of significant numbers of islets in the days following transplantation results from several factors, but ischemia and inadequate blood supply are likely contributors to islet death in the immediate posttransplant period and may impair islet survival and function long term [42]. 

Islet viability during culture is also adversely affected by hypoxia to the cells in the inner core of islets [43, 44]. Although it may be difficult to prevent a hypoxic condition of the inner islet cell mass during *in vitro* culture, genetic modulation of islets to express genes that promote rapid revascularization upon transplantation and reduced culture time could play an important role in preventing hypoxic damage to the islets [45].

Molnár and colleagues recently demonstrated that even mild islet hypoxia causes significant functional impairment of glucose-induced insulin release. In comparison with islets cultured in normoxia, insulin release is reduced by 50% already in islets cultured at a *p*O_2_ of 27 mmHg and by 98% in islets cultured at a *p*O_2_ of 5 mmHg [46]. The present findings, with formation of an extensive intra-islet capillary network after intraportal clinical islet transplantation and with only transient islet graft hypoxia (*p*O_2_ < 10 mmHg) in experimental islet transplantation, are in accordance with the capacity of an islet graft to respond with insulin secretion in response to glucose, repeatedly shown in numerous clinical and experimental islet transplantation studies [46].

One possible explanation for the requirement of islets from at least two pancreata to achieve insulin independence is that many islets die in the first days after transplantation, before adequate vascular supply is reestablished. Various studies have found that islet cell survival, islet insulin content, and cell mass declined 1–3 days after transplantation. This is the period when the islet graft is avascular.

Immediately after transplantation the islet depends on diffusion of oxygen and nutrients from the surrounding microenvironment for their survival and function. In order to regain proper islet function, new capillaries and blood vessels have to form, rebuilding their old capillary networks [1, 47]. As previously stated, the new networks are derived from both the recipient blood vessels and the remnant donor islet endothelium [39]. This revascularization process may initiate as soon as 1–3 days after transplant and may conclude around day 14 [38, 39]. 

Pancreatic islets implanted intraportally to the liver become lodge in distal tributaries. However, the new vascular network in the islets seems instead to be connected to the hepatic arterial tree [27, 48]. Because newly transplanted islets mostly likely lack nerves and it is uncertain if any functional reinnervation occurs, the islet graft blood flow regulation will largely depend on locally produced vasoactive mediators [48]. 

A striking observation is that although new blood vessels form within transplanted islets, the resulting vascular density is chronically lower than the native islets. This is irrespective of whether the islets are implanted as aggregates to the kidney or spleen or infused through the portal vein into the liver. The vascular density is not influenced by hyperglycemia or engraftment time but numerous vessels do form in the surrounding connective tissue [3]. 

A recent study successfully proved the impaired revascularization of islets within the liver [49]. They demonstrated that pancreatic islets transplanted intraportally into the liver have a very low blood perfusion, reflecting few and dysfunctional blood vessels. Donor islet endothelial cells mainly disappear or migrate into surrounding liver parenchyma; therefore, disruption of islet integrity is pivotal to support revascularization by recipient blood vessels [49]. 

The impact of the gene expression of angiogenic factors and their receptors on the revascularization of islets graft is still under investigation. However, the resulting vascular density does not differ between islets transplanted into a normoglycemic or hyperglycemic environment. Moreover, immune response does not seem to affect the revascularization process, although later on destruction of the capillary network occurs as a consequence of microvascular rejection [50].

Although transplantation in highly perfused organs such as the liver promises to provide adequate tissue bathing to provide nutrition by diffusion, the cells in the inner core of the islets still do not receive an adequate supply of oxygen and nutrients. These cells depend on intra-islet capillary-mediated flow of blood. This limitation leads to lower oxygen and nutrient supply in the inner core of islets, which constitutes predominantly the insulin-secreting *β*-cells, and ultimately leads to hypoxia and cell death. This phenomenon was elegantly demonstrated by Vasir et al., who stained islets cultured for 24 and 48 h with propidium iodide and calcein-AM to demonstrate the progressive loss of islet viability in the center of the islets [51].

As previously mentioned several authors agreed that donor endothelial cells might contribute to islet graft revascularization [3, 38, 42]. Unfortunately, endothelial cells disappear during the culture phase. Based on these findings, recent studies suggested that the lack of culture phase, and hence the use of “fresh” islets for transplantation may improve the vascularization ratio and eventually the engraftment results [46]. This phenomenon may be explained, in part, by preserved FGF excretion in noncultured islets, which has been reported to improve blood vessel stability [46]. These findings are based on syngeneic transplantation models, where revascularization can be studied in a standardised manner without interference by factors such as immunosuppression and immunological rejection. The clinical importance of these results needs to be further evaluated in the human allogeneic setting [46].

### 4.2. Strategies to Increase Revascularization

The revascularization of transplanted islets might be enhanced or accelerated by several types of interventions: increasing the action of proangiogenic agents or to inhibit antiangiogenic factors and thus stimulate the proliferation, migration, and maturation of endothelial cells into functional vessels. This analysis may be partially correct [3, 52], but it is likely that the optimal formation of mature, fully functional islet vasculature will require precise control of the timing, dose, and duration of angiogenic factor action in the posttransplant period. A second approach could directly target endothelial cells or enhance their ability to form mature, functional vessels and might involve the addition of preactivated endothelial cells or some type of endothelial progenitor cell population. These two approaches should be applicable to isolated islets before transplantation or could be used to prepare the transplantation site before transplantation of isolated islets. Finally, Johansson et al. [53] proposed a new approach using tissue engineering to enhance islet revascularization. These investigators provided evidence that the coculture of MSCs and endothelial cells with human islets *in vitro *before transplantation initiated formation of vessel-like structures that may promote islet engraftment after transplantation. MSCs, multipotent cells usually isolated from bone marrow but also present in other tissues, exhibit a wide range of properties in other settings, properties that might enhance islet survival [3, 53]. For example, MSCs positively modulate inflammation, tissue regeneration, and immune attack either through cell-to-cell contact, differentiation into other cell types, or by the local production of factors such as platelet-derived growth factor.

A recent study also found a direct association between the regeneration of liver tissue and the islet engraftment, intraportally. After partial hepatectomy, many growth factors such as HGF and VEGF-A are upregulated for regeneration in the remnant liver [54]. It is known that these growth factors have properties to promote vascularization, and therefore the authors hypothesized that revascularization of transplanted islets was enhanced during liver regeneration after partial hepatectomy [54].

The inhibitory effects of rapamycin, a key component of the immunosuppressive regimen in the Edmonton protocol, on tumor angiogenesis or pancreatic islet revascularization have been clarified [55, 56]. However, the effect of tacrolimus, which is one of the standard immunosuppressants in both pancreatic islet transplantation and whole pancreas transplantation, on revascularization was only recently elucidated [57]. It appears to inhibit the revascularization of isolated pancreatic islets without affecting the characteristics of the transplanted grafts [57]. Further refinements in this immunosuppressive regimen, especially with regard to the revascularization of islet grafts, could therefore improve the outcome of islet allotransplantation.

## 5. Hepatic and Alternative Transplant Sites

**Figure 1:**
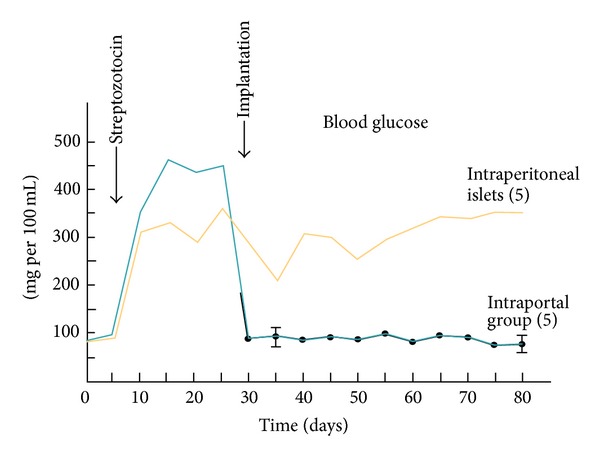
Islet transplant outcomes comparing two different sites in diabetic rats (adapted from Kemp et al. [58]).

Kemp and colleagues from Lacy's group were the first to explore the liver and intraportal site for islet transplantation in rats in 1973 [58]. In a small study of 5 rats per group, they compared intraportal islet implantation with intraperitoneal implantation and found that diabetes was reversed only when islets were implanted into the portal vein (Figure 1). This study had profound impact on the translational development of clinical islet transplantation, where almost universally islets have been implanted into the hepatic portal vein in over 1,085 islet transplant patients, according to the most recent report of the Collaborative Islet Transplant Registry [59]. Where other sites have been attempted in patients, these have as yet never rendered patients insulin independent. Thus, although intraportal islet transplantation has empirically been accepted as the best site to use in patients, we herein briefly review the evidence to support this and compare potential alternative sites for future clinical development.

### 5.1. Intraportal Site

The portal vein is far from the ideal infusion site with half of infused islets dying shortly after transplantation [60]. In addition, over time most patients resume using insulin injections. Portal vein infusion results in embolization of islets in the liver that exposes the cells to a relatively hypoxic environment since the liver has a parenchymal oxygen tension below that of the pancreas [61, 62]. Furthermore, infusion into the portal vein exposes patients to additional risks of hemorrhage, thrombosis, biliary puncture, transient rise in serum aminotransferase, and arterial-venous fistula. Since native islets deliver insulin directly into the portal vein, it follows that the best method to mimic normal endogenous release would be to infuse islets into this site. However results from whole pancreas transplantation showed that when portal venous drainage for the transplant was utilized there was limited metabolic benefit in comparison to systemic drainage [63], suggesting release of insulin directly into the portal vein is not essential. 

Despite the problems with portal vein infusion, it still accounts for 90% of clinical islet transplantations. The liver has been shown to play a key role in regulating systemic insulin levels, and hence delivery of secreted insulin directly to the liver is ideal for maintaining tight glycemic control [64]. This was further illustrated by intraperitoneal infusion of insulin which led to delayed systemic distribution of insulin in comparison with intraportal infusion [65]. The portal vein also appears to be more economic in islet uptake since fewer islets are required to reverse diabetes compared with other transplant sites. Studies in rats showed that only 550 autologous islets were required to reverse diabetes with portal vein infusion compared with a partial reduction in hyperglycemia with 770 islets infused into the peritoneum and a failure of any hyperglycemia reversal with 890 islets infused into a subcutaneous site [58]. Subsequently, the portal vein has become the standard for comparison with other transplantation sites. 

### 5.2. Improving the Intraportal Site

The portal vein and for that matter all vascular transplant sites undergo instant blood-mediated inflammatory reaction (IBMIR) which results in an early inflammatory reaction [60]. IBMIR limits *β* cell function after transplantation, and therefore it is essential to avoid this by either identifying a transplant site with minimal interaction with blood or by protecting vascular grafts from IBMIR. Currently there are a number of strategies aimed at preventing IBMIR including using nicotinamide [66], low molecular weight dextran sulfate [67], thrombin inhibitor [68], and heparin coating islets [69]. Despite these strategies, IBMIR remains a limiting factor on *β*-cell function with the intraportal site in addition to other vascular sites. 

### 5.3. Renal Subcapsular Site

In rodents, the renal subcapsular site is the most widely used transplantation site. Practically, it provides a readily accessible site; functionally, it reverses hyperglycemia within days of transplant; and histologically, investigation is easily achievable by recovery nephrectomy. Less than 25% of normal islet mass is required in the renal subcapsular site to maintain normoglycemia [70], and in immunodeficient mice, this site is better than the lung, liver, or spleen for functional engraftment [71]. However as with the liver, the renal capsule is a relatively hypoxic environment (15 mmHg O_2_) in comparison to pancreas parenchyma (40 mmHg O_2_). Studies comparing the renal subcapsular site with the intraportal site in mice indicate that a much smaller number of islets are required in the subcapsular site (250 islets) to reverse diabetes in mice versus the portal site (700 islets) [72, 73]. This apparent superiority of subcapsular islets in mice is likely related to the differences in islet preparations between mice and humans. Firstly, mice islets are larger and less fragmented that those in human preparations, and secondly, due to a smaller portal vein diameter in mice, islets embolize earlier in the vascular tree resulting more hepatic necrosis and reduced blood supply to the intraportal site [74]. 

Clinical studies using this kidney site for islet transplantation resulted in C peptide secretion in two of three diabetic patients. However, a high transplant mass was required at this site in comparison with the intra-portal site rendering this site inferior [75]. Furthermore, from a surgical standpoint the renal subcapsular site provides difficult access for infusion in humans being very invasive, and the presence of diabetic nephropathy in a large proportion of the recipients reduces the efficacy of this site. Although the renal subcapsular site provides an attractive experimental model in mice it has no clear gain in humans.

### 5.4. Spleen

Infusion of islets into a splenic vein tributary and directly injecting islets into the splenic pulp have both been proposed as potential islet transplant strategies. In a canine model, autotransplant into splenic sites resulted in a similar reversal of diabetes as that seen with hepatic sites [76]. Despite being a metabolically suitable site for islet transplantation with promising results seen in large mammals [77–80], the spleen offers no advantages over the liver. The patient is exposed to an added risk of hemorrhage from splenic rupture, and the transplanted islets are more readily accessible by lymphocytes making it a poor potential site.

### 5.5. Omentum

The peritoneum offers an unlimited space for transplanted islets and therefore offers an attractive site for concurrent use with encapsulated devices to protect the islets [81, 82]; however recovery of these islets for histological and functional assessments is difficult [83]. In rats, at least 1500 islets were required to reverse diabetes, and due to a lack of parasympathetic reinnervation at this site, abnormal glucose tolerance tests were noted [84]. 

Surgically creating a pouch using omentum and parietal peritoneum provides a site for islet transplantation with an increased vascular supply [85]. In diabetic rats an omental pouch required 2000 islets to reverse the diabetes with the resulting normoglycemia lasting more than 6 weeks [86]. The high vasculature observed within the omentum and the presence of proangiogenesis cytokines [87] along with the immune-privilege provided by the peritoneum [88, 89] makes this an attractive site. However with the large islet numbers required and limited long-term function shown in current studies, further development of this site is required for it to progress to clinical use. 

### 5.6. Pancreas

Being the native home of islets, the pancreas has long been suggested to be an optimal site for islet transplantation. In mice, islets recovered from a pancreatic site were metabolically superior than those reisolated from the intraportal site [90] suggesting that the pancreas may provide a more optimal site for long-term islet function. Fewer islets were required to reverse hyperglycemia in rats with the pancreas site (500 islets) compared with the portal site (3200 islets) and the renal subscapular site (2000 islets) [91]. These superior results observed with the pancreatic site have been attributed in part by the accurate reproduction of the native islet environment with regard to oxygen partial pressure, glucose detection, and insulin release. Despite this, the presence of preexisting type 1 diabetic may make the pancreas a poor site since pancreatic lymph nodes may be more primed and equipped to promote a rejection episode. Furthermore, the infusion process is invasive and would carry substantial risk in humans through risk of inducing severe and life threatening complications from pancreatitis. Additionally, in patients undergoing pancreatectomy with autotransplantation, this site becomes inappropriate. 

### 5.7. Gastrointestinal Wall

The wall of the gastrointestinal tract is the natural entry site for glucose into the body and consequently is an ideal location for islet cells to sense glucose. The accessibility to the submucosa via endoscopy, the highly vascular mucosa, and the bioavailability to oral therapeutic agents makes this an appealing site. Hamster [92], rat [93], and pig [94] models have illustrated efficacy with the gastric submucosa and subserosal sites showing them to be superior to the renal subcapsular with regard to glycemic function; however as yet there is no comparison to the portal site. 

### 5.8. Immunoprotected Sites

The thymus, brain, and testis may provide protection from the recipient's immune system with an allotransplant thereby potentially reducing the requirement for simultaneous immunosuppressive agents. Islet transplantation into the testis was successful in controlling diabetes in rats [95, 96] and delayed rejection with allografts [89, 97]. Sertoli cells, which provide the blood-testis-barrier, have also been utilized at other transplant sites. Autologous sertoli cells cotransplanted with allogeneic islets under the renal capsule improved normoglycemia compared with islets alone [98] and delayed rejection even in the absence of immunosuppression [99, 100]. We await results from large animal testicular islet transplants and sertoli-islet cotransplants to see if such immunomodulatory approaches can be translated from mice to humans.

The cerebrum [101] and cisterna magna [102] have both been shown to attenuate hyperglycemia and delay the onset of allograft rejection. However the risk with the brain transplantation site makes this an improbable clinical prospect.

The thymus has been studied as a transplant site in rodent, canine, and porcine models and has now entered clinical studies. The organ is easily accessible surgically and practically makes an attractive transplant site. In diabetic rats, allogeneic islet infusion along with a one off dose of antilymphocyte serum led to normoglycemia for over 6 months and induced tolerance of further islet infusion under the renal capsule [103]. Theoretically, since the thymus is the site of T-cell maturation, maturing T cells will be exposed to islets enabling negative selection of reactive T cells toward the islet alloantigens thereby resulting in selective deletion of islet-specific T cells. Indeed, an autoimmune model of type 1 diabetes in rats showed long-term survival of intrathymic transplanted islets [104]. Despite this promising immunological theory, the thymic site requires a large number of islets to reverse hyperglycemia [105–107]. 

### 5.9. Musculoskeletal Sites

The bone marrow of rats has been revealed to be a potential site for islet transplantation with insulin histological studies showing persistence of insulin-containing cells 3 weeks postallogeneic transplant [108]. Another study using syngeneic islets transplanted into the bone marrow reversed hyperglycemia for greater than 1 year in diabetic mice and showed a 2.4-fold increase in euglycemia versus transplantation via the intra-portal site [109]. Further work is required on both long and short bone sites before this potential location can be utilized in the clinic.

Muscle is easily accessible and can be readily biopsied making it an attractive site. Indeed after transplant into humans, biopsy illustrated *β*-cell staining in two of three patients; however this was associated with a large leucocytic infiltrate [110]. The epididymal [111] and mammary [112] fat pads in mice have also been subject to islet cell infusion. In both studies, only a small volume of islets was required to reverse hyperglycemia, and it was speculated that the improved vasculature of adipose makes this a particularly exciting treatment avenue. Musculoskeletal sites are easy to access, offer substantial space in which to transplant cells, and are highly vascularized making them a very welcoming area for future research (Table 1).

### 5.10. Subcutaneous Site

Subcutaneous macroencapsulated islets transplanted into humans illustrated *β*-cell survival and glucose-dependent insulin secretion two weeks after implantation without immunosuppression [113]. Microencapsulated islets within a prosthetic device connected to blood vessels showed reversal of hyperglycemia in one of two recipients with both patients showing positive C-peptide after transplant [114] (Table 1). 

Despite the diversity of the transplant sites that have been investigated to date, as outlined above, their ability to successfully promote islet graft survival is linked to a common ability to foster revascularization. Using this rationale, our laboratory is currently investigating whether a previously suboptimal, low oxygen tension, transplant site can be naturally manipulated to become an appropriate surrogate for islet engraftment. By utilizing the natural foreign body response, to our “deviceless” technique we have been able to transform the subcutaneous site into a highly vascularized transplant site, rich in islet supporting microvessels, leading to long-term islet graft function. 

## 6. Summary

Islet revascularization appears to be the critical component in ensuring islet survival and function, irrespective of the transplant site. To date, despite promising research into alternative engraftment strategies, few have translated into the clinical setting. The gold standards for islet transplantation in the clinical and experimental settings remain the intrahepatic portal infusion and kidney capsule, respectively. Unfortunately studies have indicated that islets transplanted intraportally have hindered abilities to become revascularized, highlighting the need for additional extrahepatic transplant research. Here we have described that an optimal engraftment site requires access to adequate oxygen and nutrient supplies whether from endogenous vasculature or from induced or intrinsic neovascularization, in addition to a supporting matrix or scaffold. Furthermore, graft retrievably appears to be an important consideration when testing alternative transplantation sites, especially when considering their potential to house insulin producing stem cells. Since engraftment is governed largely in part by revascularization, there appears to be endless opportunities to formulate adequate alternative transplant strategies, with the caveat that the engraftment approaches result in the islets being close proximity to a vascular-rich matrix. Taken together, it appears that continued research in the areas of islet revascularization and engraftment holds great promise in advancing the therapeutic benefit of islet transplantation. 

## Figures and Tables

**Table 1:** Characterization of different islet transplant sites as published in the literature.

Site	Local environment challenges	Glyco-insular response	Portal versus systemic insulin delivery	Immune exposure	Surgical accessibility	Preclinical evidence	Clinical evidence
Intraportal	++	+++ Mimics physiological insulin release	?	0 IBMIR	+++ Minimally invasive	[58, 74, 76, 81, 91, 105, 115–117]	[118–120]
Renal subcapsule	+ Hypoxia	0 Need many islets	Systemic	++	0 invasive	[74, 76, 91, 115, 116, 121, 122] Good rodent evidence	[75]
Omental pouch	+ Hypoxia	+	Portal	++	++	[85, 123, 124]	
Gastrointestinal wall	++	+++ Physiological glucose entry site	Portal	++	+++ Endoscopic access	[92–94]	
Subcutaneous	+ Prevascularization required	+	Systemic	+	+++	[60, 125–127]	
Muscle	+ Prevascularization required	+	Systemic	+	+++	[125, 128–130]	[110, 131]
Bone marrow	+++ Highly vascular	+	Systemic	+	0 Invasive	[108, 109, 132]	
Adipose	++ Vascular	+	Systemic	+	+++	[111, 112]	
Pancreas	+++ Native site	+++ Native site	Portal	+	0 Invasive	[91, 133]	
Spleen	+++ Highly vascular	+++	Portal	0 IBMIR	0 Hemorrhagic risk	[76, 134–137] Good canine evidence	[124]
Lung (intravenous)	+ Venous supply is hypoxic	++	Systemic	0 IBMIR	0 Widely dispersed	[138, 139]	
Brain	+++ Highly vascular	++	Systemic	+++ Immune-privileged	0 Cerebral ischemia risk	[101, 102]	
Testis	+	+	Systemic	+++ Immune privileged	++	[97, 98, 140]	
Thymus	+	+	Systemic	+++ Immune-privileged	++	[104–107]	[141]
Celiac artery	+ Infarct in terminal end-arteries	++	Portal	+ IBMIR	+	[117]	

+++: excellent ++: good +: neutral 0: disadvantage.

## Abbreviations

IAPP: Islet associated polypeptide
IBMIR: Immediate blood mediated inflammatory response
MSCs: Mesenchymal stem cells
VEGF-A: Vascular endothelial growth factor A
HGF: Hepatocyte growth factor
FGF: Fibroblast growth factor
EGF: Epidermal growth factor
MMP: Matrix metalloproteinase.
